# Medical News and Miscellany

**Published:** 1887-11

**Authors:** 


### Medical News and Miscellany.

Cincho Quinine and a "fruit diet,' will often relieve aggravated cases of malarial poisoning.

---

We learn that by a Cablegram, London, October 25, W. R. Warner & Co., Philadelphia, received highest reward from American Exhibition in London for superiority of their Sugar-coated Pills and Effervescing Salts.

---

### ANTIPYRINE.

It is not generally known that antipyrine is a "patent medicine." Such is a fact, however; it was patented in Europe and America, in '84—and, under the laws of France, its use is prohibited in that country.

---

The Crown Prince of Germany's disease has at last been acknowledged by his doctors to be, what every body else believed all along, cancer; and at last accounts the great professors from all parts of Europe had been summoned in consultation, with a view, it is supposed, of considering the advisability, or necessity, of the entire removal of the larynx, a last and desperate resort.

---

### DIED.

Dr. J. McKenzie Johnson, County Physician of Galveston county, died in Galveston Wednesday evening, November 9, aged 32 years. Dr. Johnson was a native of Galveston county, and had held the position of County Physician four years. He was very popular, and was a prominent member of the order of Knights of Pythias.

---

### OUR PARIS LETTER.

We call attention to Dr. Weymann's letter from Paris, and take pleasure in informing our readers that we have made arrangements with the Doctor for a letter each month. He will visit the principal cities and hospitals of Europe, and will furnish this Journal with short, crisp and newsy letters, giving accounts of all that is new and interesting in medicine and surgery, as practiced by the big guns of the old world.

THE CROWNING ACHIEVEMENT OF OPHTHALMIC SURGERY OF THE PRESENT CENTURY.

Under this title Dr. L. Webster Fox describes in the *Medical and Surgical Reporter*, Professor von Hippel's operation for the transplantation of the rabbit's cornea to that of man. By this operation Von Hippel has restored sight to an eye practically blind, and Dr. Fox predicts that it has a brilliant future in doing this kind of work. He saw the patient upon whom the first successful operation was performed, and learned its technique. We cannot do better than furnish to our readers Dr. Fox's very clear and complete description.

The patient operated upon was a young, healthy peasant girl, nineteen years of age, who suffered with a leucoma of the cornea of the right eye, obscuring vision to such an extent that qualitative perception of light ouly remained. The leucoma simplex obscured the central portion of the pupil, leaving, however, a circle of clear cornea at its outer margin, probably two mm. wide. The instrument devised by Prof. von Hippel for performing this operation is most ingenious. The trephine is driven by a clockwork and the cylinder is graduated so as to regulate its cutting depth in the cornea. The leucomatous cornea opposite the pupil was removed by this instrument. The eyelids were separated by an ophthalmostat; then, under the influence of cocaine, the trephine was placed on the cornea perpendicular to its plane, the cylinder so graduated as to cut a certain depth, 0.7 to 0.9. This cylinder is then put in motion by a spring clock motion much after the manner of a Dudgnon's sphygmograph; the hand simply steadies the instrument against the probe; after the circular incision is made comes the most important and delicate part of the operation, *i. e*, the dissection of the leucomatous tissue from Descemet's membrane. This is done by grasping the inner lip of the incised tissue, and with the greatest care and precision this tissue is removed down to the basement membrane lying in juxtaposition to Descemet's membrane. If, after the removal of this circular piece of cornea, it is found that Descemet's membrane bulges forward through the circular opening, which in almost every case it does, a paracentesis of the anterior chamber is made at the corneoscleral margin, relieving the intra-ocular pressure.

The rabbit selected from which to obtain the graft is a young, healthy doe. The eye, which is thoroughly cocainized, is drawn forward by an assistant who has inserted under the superior and inferior recti muscles two strabismus-hooks; the eyelids are kept open with an ophthalmostat; the drawing forward of the globe enables the trephine to be inserted and watched more accurately in its incision; the cut is made through cornea and Descement's membrane; this graft is then inserted in the incision made in the patient; a fine probe running through the cylinder of the trephine is pushed downward, forcing the graft into place; after the removal of the trephine the upper eyelid, which is drawn forward and downward, is pressed against the inlaid tissue, all being held firm by a pressure-bandage, delicately adjusted, the patient, of course, lying on his back. After three days the bandage is removed, and the eye examined. If the graft is *in situ*, it will probably be somewhat hazy; if the edges have not turned upward, a successful result may be prognosticated.

Dr. Hippel recently showed a second patient and demonstrated his operation before the Ophthalmological Society of Heidelberg. The patient was found to have a visual acuity of 20-200, and read Jaeger's No. 6, from which it would be inferred that the new cornea did not clear up completely.—*N. Y. Med. Record.*

---

### THE STENOCARPINE SENSATION.

### AN ATTEMPT TO IMPOSE UPON THE MEDICAL PROFESSION.

Our readers are doubtless familiar with the reports of Drs. J. H. Claiborne, Hermann Knapp and Edward Jackson, concerning the so-called new local anæsthetic, Gleditschine or Stenocarpine, which were published in the *New York Medical Record*, July 30th, August 13th and October 1st; and *Philadelphia Medical News*, September 3rd; in which Gleditschine was claimed to possess remarkable anæsthetic and mydriatic properties.

It will, therefore, be of interest to them to learn that Messrs. Parke, Davis & Co. announce that an investigation at their laboratory, of a solution purporting to be a 2 per cent. solution of Gleditschine, or Stenocarpine, which was supplied by Messrs. Lehn & Fink, of New York, has developed the fact that this solution, with which the experiments thus far recorded have been made, contains 6 per cent. of Cocaine and a sulphate of a salt which further experiment is likelp to prove to be Atropia.

F. A. Thompson, Ph. C., also reports, after careful experiment with the leaves of Gleditschia triacanthos, from which Gleditschine or Stenocarpine is claimed to have been derived, that they contain only an infinitesimal percentage of an amorphous alkaloid devoid of anæsthetic or mydriatic properties.

In the light of these facts it seems probable that the Stenocarpine sensation should be classed with the Hopeine fraud of malodorous memory, and that the physicians who have already published reports regarding Gleditschine or Stenocarpine have been the victims of a clever hoax. (*By request.*)

---

### BOOK NOTICES.

Owing to the illness of the editor during the first half of November, no book notices have been prepared, and quite a number of books are awaiting review. We ask the indulgence of publishers

---

### AUSTIN SANITARIUM.

This institution was organized on the 7th inst., the Board of Directors consisting of Dr. T. J. Tyner, Dr. J. W. McLaughlin, Dr. R. Steiner, Dr. B. E. Hadra, and Mr. Walter Tips. Dr. Tyner was elected President, and Dr. Hadra Manager and Secretary, and Mr. Tips, Treasurer. A charter has been secured, and the stockholders embrace many of the wealthiest and leading business men of Austin. The institution will be for females only, and for surgical diseases. A large building has been secured and splendidly equipped; and patients will find every comfort prepared for them; while the names of the distinguished physicians having charge is sufficient guarantee that they will receive the best attention and highest medical and surgical skill. It is expected that this institution will become a credit to the proud Capital City of Texas.

---

### ACQUITTED.

Dr. G. S. Felder, of Webberville, Travis county, who had been enticed, while under the influence of drink, into a den where he was being fleeced, killed one of the crowd (a colored man), last winter, after a patient and tedious trial, was unanimously acquitted last term of court. Dr. Felder is a promising young physician, and his friends, who sympathised with him in his misfortune, now rejoice with him in his acquittal.

---

Lehn & Fink, the enterprising chemists of New York, who charged Park, Davis & Co. six dollars for an ounce mixture purporting to be a two per cent. solution of the alleged newly discovered alkaloid *gleditschine*, which had become notorious as a rival of cocaine, in consequence of published reports by Drs. Claiborne and Knapp, and which solution, on analysis, proved to be a six per cent. solution of cocaine, and worth about fifty cents, *now* claim that "Mr. Goodman," the alleged discoverer, had placed a quantity of the solution, in bottles, in their hands for sale. (See Druggist's Circular, November, page 262.)

Too thin. Dr. Claiborne, in his article in *Gaillard's Journal* for November, says the solution he had, and with which he made the experiments, was all there was of it; and we understand that it was made by Goodman at Bayou Sara, La., extemporoneously.

A paragraph on the next page of the Druggist's Circular, announces the *arrest of Lehn & Fink* on a charge of *counterfeiting Merk's labels.* This, probably, furnishes a solution to the question But. notwithstanding the entire reliability and honesty of Drs. Claiborne and Knapp, there is still a mystery about the subject. Park, Davis & Co.'s pharmaceutical chemist, Dr. Thompson, was unable to find any of the alkaloid in a lot of leaves and twigs of the tree, acknowledged to be the source from which Goodman claims to have procured it. The trouble seems to be that Goodman (by the bye, who, besides being "a veterinary surgeon," is Mr. Goodman ?), wants to make money out of his "discovery;"—wants to keep the process secret by which he claims to have eliminated the alkaloid, so-called, notwithstanding his apparently fair offer as reproduced in this number from the New York *Medical Record.*

There is something rotten in Denmark—and it begins to look like a collusion between Goodman, and Seward, and Lehn & Fink, and that Drs. Claiborne and Knapp have been unmercifully duped.

### THE NACOGDOCHES TEST CASE.

Physicians of Texas will read with interest the report of the test case in Nacogdoches county, and will await with anxiety the decision—as it will establish a precedent which will doubtless settle this vexed question. It seems there is much difference between *law* and *justice,* sometimes. It is, to us, absurd that a physician should be put upon the stand, and called a witness, and, if paid at all, paid as a witness, when, instead of being asked to testify to facts, he is made to *give an opinion.*

To acquire the knowledge requisite to give that opinion value, the physician has expended much time and money, and his knowledge constitutes, really, his *stock in trade,* his goods and chattels; and there would be as much *justice* in taking merchandise by force, as in extorting an opinion from a physician, under the absurd pretext that he is "a witness." He is, in no sense, a witness. A witness testifies to what he knows, and is not permitted to give an opinion, or to say what he thinks; whereas a physician is asked, not what he knows, but what he thinks—his opinion.

The medical profession should arise as one man, and protest against such robbery; and Dr. Fears deserves their thanks for having the courage to test the matter. If it should be decided that a physician can be made to give his services at inquests, at laborers' wages, and to give "expert testimony"—*his opinion*—for $1.50 a day, soon it will be difficult to get a physician to attend a case likely to get into court. Instead of the government policy whereby the co-operation of the medical profession can be had in the enforcement of the laws, the treatment physicians receive before the courts is calculated to alienate them; and they will get out of the way of a summons, and withhold information which may be very valuable to the ends of justice. No other class of people are subjected to such an outrage. Why should a physician be expected to do more for his country than any one else?

---

### A CURE FOR WRINKLES.

Wool-fat, or agnine, is made from the wool of sheep, by steeping the clippings in hot alcohol. By this process a yellow grease is precipitated, chemically identical with an element found in the human oils, and in certain vegetables, such as peas and beans. This grease has recently been found to have one very peculiar property which was accidentally discovered by Dr. Morton Prince, of Boston. When applied with rubbing, it passes directly through the skin, and in this way acts as a nutrient to the fatty tissues beneath. Thus, it has the effect of smoothing out the wrinkles produced by the attenuations of these tissues which come with age. An antiquated lady has nearly removed from her temples the unwelcome footprints of a thousand figurative crows, by six weeks use of it. Dr. Prince has also used it with success in other cases, and it has created quite a sensation in Boston.—*Druggists Circular.*

---

### DEATH OE PROFESSOR MOSES GUNN.

Professor Gunn, of Rush Medical College, Chicago, one of the most celebrated surgeons west of the Allegheny mountains, died in Chicago, Nov. 4, of some chronic, or malignant, disease of the liver. Dr. Gunn was a "natural surgeon," and was said to be gifted with the ability to make a correct diagnosis almost instinctively. He was a most scrupulous observer of professional etiquette, and was remarkable for his considerate deportment towards all professional men; hence, he was immensely popular. His position in Chicago was what Gross' was in Philadelphia, and Stone's in New Orleans—*the* surgeon, universally looked up to, and respected by all classes, and his ability recognized and deferred to by his colleagues. His loss will be a serious set back to the Rush Medical College.

---

### A DOCTOR IN LIMBO.

Prof. StJohn, of the Chicago College of Physicians and Surgeons, was recently indicted for complicity in the escape of the "boodle alderman," McGarrigle. We do not know the penalty, nor the particulars.

---

### GAILLARD'S MEDICAL JOURNAL.

Dr. P. B. Porter has retired from the editorial management of this popular publication, and hereafter it will be edited by the owner and publisher, Mrs. M. E. Gaillard, assisted by Geo.Tucker Harrison, M. A. M. D. and J. Herbert Claiborne Jr., M. D., both Virginians. The management is to be congratulated on its excellent corps of collaborators, which consists of T. Gaillard Thomas, M. D., W. T. Lusk, M. D., D. Bryson Delavan, M. D., and P. B. Porter, M. D., of New York, and Hunter McGuire, M. D., of Richmond, and C. H. Mastin, M, D., of Mobile. The first number of the journal under the new management is bright and attractive in its make up, the contents embracing a variety of interesting original and selected matter.

Mrs. Gaillard deserves, and we are happy to say, is receiving a cordial support from the profession of America; her enterprise and pluck have challenged the admiration of the profession everywhere, and, the distinguished gentlemen whose names appear on the cover of her journal have gallantly come to her aid and support from pure admiration for, and sympathy in her heroic endeavors to keep the Journal up to its old time standard of excellence; aud declining any compensation, give their names and their services to this end with a chivalry which has ever characterized the Virginia gentleman. We cordially endorse the *Journal* as "the best journal with which we are acquainted," and recommend our readers to subscribe for it, if they would get the best and purest medical literature of the day.

---

BIOGRAPHY OF CONTEMPORARY PHYSICIANS OF TEXAS, ILLUSTRATED.

Those who have not yet contributed the data from which to prepare biographical and sketches for this work are requested to communicate with the publishers, S. H. Dixon & Co., Austin. They request us to give notice that the work is to be pushed to completion as soon as the financial condition of the country will enable them to complete the subscription list, and also to say that arrangements have been made to secure electro-engravings at a price much lower than at first announced. A good cabinet photo should be sent. The terms are easy.

In a paper read before the Surgical Section of the International Medical Congress, held at Washington, D. C., on Sept. 5, 1887, styled, "When is Colotomy Justifiable?" Joseph M. Matthews, M. D., Professor of Principles and Practice of Surgery, and Clinical Lecturer on Diseases of the Rectum at the Kentucky School of Medicine, said, " I can not understand why anyone would advise Colomy in cases of ulceration, per se, of the rectum. With strict antiseptic precautions, the rectum can be kept perfectly clean, and with the aid of the different meat extracts and fluid foods in the market, the bowel can be absolutely rested any length of time.

The milk as recommended by Mr. Allingham can be used, or what is better than all, in my experience, the preparation called Bushes' Fluid Food, or *Bovinine*, which contains 26.58 of soluble albuminoids, and is the vital principle of beef, obtained by a new process. It is a raw fluid food extract, and admirably suited to those cases which require, during treatment, entire absence from the solids. I have kept patients for weeks on this preparation alone, during which time local applications were made to the bowel, until all ulceration had healed."

